# Retraction: Simultaneous Inhibition of mTOR-Containing Complex 1 (mTORC1) and MNK Induces Apoptosis of Cutaneous T-Cell Lymphoma (CTCL) Cells

**DOI:** 10.1371/journal.pone.0239102

**Published:** 2020-09-10

**Authors:** The PLOS ONE Editors

After this article [1] was published, concerns were raised about results reported in Figures 2 and 3A.

Specifically,

- Similarities were noted in Figure 2E between lanes 1 and 2 of the CTCL p#1 eIF4E S209 panel and lanes 1 and 2 of the CTCL p#1 ERK1/2 T202/Y204 panel. The relative intensities of bands in lanes 1 and 2 is different between these two panels, and there are some differences in pixilation in the background regions of the two panels. However, the band shapes and patterns of pixilation intensities within the corresponding bands appear more similar than would be expected for different blot results.
- There appear to be vertical discontinuities in the background after lanes 1, 2, and 3 in the eIF4E S209 panel in Figure 2E, and after lane 3 in the ERK1/2 T202/Y204 panel of Figure 2E.
- In the ERK1/2 T202/Y204 blot of Figure 3A, lanes 1, 3, and 8 appear similar and lanes 2 and 7 appear similar. There also appear to be vertical discontinuities in the image after lanes 1, 3, 4, 5, and 6.

In response to the concerns about similarities between lanes in Figure 2E and 3A (first and third points, above), the corresponding author noted that the bands in question are negative controls that are expected to match one another.

In response to the first bullet point, the corresponding author commented that the differences between the two panels as described above provide evidence that they report different data. The authors provided cropped electronic images of the Figure 2E blots, but these did not fully resolve the concerns.

The authors stated that the original raw data for the experiments in question are not available.

In light of the above concerns which call into question the reliability of the results reported in Figures 2E and 3A, the *PLOS ONE* Editors retract this article.

MW did not agree with retraction. MAW and NO disagree with retraction and stand by the article’s results and conclusions. The other authors either could not be reached or did not respond directly.
